# Analysis of amino acids, hydroxy acids, and amines in CR chondrites

**DOI:** 10.1111/maps.13586

**Published:** 2020-12-16

**Authors:** José C. Aponte, Jamie E. Elsila, Jason E. Hein, Jason P. Dworkin, Daniel P. Glavin, Hannah L. McLain, Eric T. Parker, Timothy Cao, Eve L. Berger, Aaron S. Burton

**Affiliations:** ^1^ Department of Chemistry Catholic University of America Washington District of Columbia 20064 USA; ^2^ Solar System Exploration Division NASA Goddard Space Flight Center Greenbelt Maryland 20771 USA; ^3^ University of British Columbia British Columbia V6T 1Z2 Canada; ^4^ Department of Chemistry University of California Merced California 95343 USA; ^5^ Astromaterials Research and Exploration Science Division Texas State University / Jacobs JETS Contract NASA Johnson Space Center Houston Texas 77058 USA; ^6^ Astromaterials Research and Exploration Science Division NASA Johnson Space Center Houston Texas 77058 USA

## Abstract

The abundances, relative distributions, and enantiomeric and isotopic compositions of amines, amino acids, and hydroxy acids in Miller Range (MIL) 090001 and MIL 090657 meteorites were determined. Chiral distributions and isotopic compositions confirmed that most of the compounds detected were indigenous to the meteorites and not the result of terrestrial contamination. Combined with data in the literature, suites of these compounds have now been analyzed in a set of six CR chondrites, spanning aqueous alteration types 2.0–2.8. Amino acid abundances ranged from 17 to 3300 nmol g^−1^ across the six CRs; hydroxy acid abundances ranged from 180 to 1800 nmol g^−1^; and amine abundances ranged from 40 to 2100 nmol g^−1^. For amino acids and amines, the weakly altered chondrites contained the highest abundances, whereas hydroxy acids were most abundant in the more altered CR2.0 chondrite. Because water contents in the meteorites are orders of magnitude greater than soluble organics, synthesis of hydroxy acids, which requires water, may be less affected by aqueous alteration than amines and amino acids that require nitrogen‐bearing precursors. Two chiral amino acids that were plausibly extraterrestrial in origin were present with slight enantiomeric excesses: L‐isovaline (~10% excess) and D‐β‐amino‐*n*‐butyric acid (~9% excess); further studies are needed to verify that the chiral excess in the latter compound is truly extraterrestrial in origin. The isotopic compositions of compounds reported here did not reveal definitive links between the different compound classes such as common synthetic precursors, but will provide a framework for further future in‐depth analyses.

## Introduction

Carbonaceous chondrites contain diverse suites of soluble organic compounds, including carboxylic acids (Yuen and Kvenvolden 1973), hydroxy and amino acids (Kvenvolden et al. 1970; Peltzer and Bada 1978), amines (Pizzarello et al. 1994), polyols and sugar acids (Cooper et al. 2001; Cooper and Rios 2016), and nucleobases (Stoks and Schwartz 1979; Martins et al. 2008; Callahan et al. 2011). The abundances and structural and isotopic distributions of many of these compounds have been found to vary significantly among meteorites of different carbonaceous chondrite groups, as well as between members of the same groups that experienced different degrees of secondary alteration processes including thermal and aqueous alteration (Glavin et al. 2010; Herd et al. 2011; Chan et al. 2012; Burton et al. 2012a, 2012b, 2014b; Pizzarello et al. 2012; Martins et al. 2015; Elsila et al. 2016; Aponte et al. 2017, 2019; Simkus et al. 2019). These secondary processes, or petrologic histories, of carbonaceous chondrites have been well studied, initially with the assignment of integer petrologic types ranging from 1 to 6, where type 3 chondrites experienced essentially no post‐accretion secondary alteration. Meteorites with types <3 experienced increasing aqueous alteration, and those with types >3 experienced increasing thermal alteration (Van Schmus and Hayes 1974; McSween 1979). More recently, new classification schemes have been developed to better distinguish the degrees of aqueous alteration, including a system based on phyllosilicate abundances, and hydrogen abundance and isotope composition (Alexander et al. 2013; Howard et al. 2015). A second system based on petrography and oxygen isotopic composition was also developed for CR chondrites, ranging from petrologic type 2.0 to 2.8 (Harju et al. 2014). The two scales generally agree on the relative degrees of alteration among samples, though the absolute numerical alteration types differ between them. The combination of having well‐characterized samples that experienced a wide range of aqueous alteration and high abundances of soluble organic compounds makes the Renazzo‐type (CR) chondrites well suited for studies on the effects of aqueous alteration on organics in meteorite samples with similar mineralogical composition (Martins et al. 2007; Pizzarello et al. 2008; Glavin et al. 2010; Aponte et al. 2016, 2019; Simkus et al. 2019).

In this work, we focus on three classes of soluble organic compounds: amino acids, hydroxy acids, and amines. Each of these classes of compounds has been studied previously in types 1, 2, or 3 CR chondrites (Martins et al. 2007; Pizzarello and Holmes 2009; Pizzarello et al. 2010, 2012; Glavin et al. 2010; Aponte et al. 2016), although not in a coordinated fashion. Here, we present the results of amino acid, hydroxy acid, and amine analyses of two CR chondrites previously unstudied for these compound classes, Miller Range (MIL) 090001 and Miller Range (MIL) 090657, and compare their distributions with data from other meteorites to gain insights into the synthetic histories and potential relationships between these compounds.

## Materials and Methods

### Materials

All glassware and tools that came into contact with the meteorite samples were heated in air at 500 °C for at least 18 h to remove organic contaminants. Standards and reagents were purchased from Alfa Aesar or Sigma‐Aldrich and used without further purification except as noted below. Water used at all stages of tool cleaning and sample processing was purified by either a Millipore Integral 10 UV system (18.2 MΩ cm, <3 parts‐per‐billion [ppb] total organic carbon) at the NASA Goddard Space Flight Center (GSFC) or a Millipore Advantage A10 system (18.2 MΩ cm, <5 ppb total organic carbon) at the NASA Johnson Space Center (JSC). Chemicals used during processing were purchased from Sigma‐Aldrich, Acros Organics, or Fisher Scientific, with the exception of some five‐carbon amino acid standards, the sources of which were described previously (Glavin and Dworkin 2009). We used HPLC grade dichloromethane (DCM), semiconductor grade sodium hydroxide (NaOH), and doubly distilled hydrochloric acid (HCl). Silica gels were purchased from SiliCycle (Silia*Bond*®, 40–63 µm particle size) and cleaned using methanol and DCM followed by drying under vacuum. Procedural blanks (used to quantify the concentration of amino acids, hydroxy acids, and amines present in the solvents and derivatization reagents prior to sample analyses) and a powdered serpentine sample heated to 500 °C in air (used as meteorite analog material to test the derivatization method and instrument responses) were carried through the identical extraction procedure as the meteorites.

### Meteorite Samples and Processing

Multiple samples of MIL 090001 and MIL 090657 were allocated by the Meteorite Working Group and provided by JSC for this study. For MIL 090001, a mass of 317 mg (designated by JSC curation as parent 4, specific 16) was processed for amino acid analysis, after which an additional 18 g (parent 4, specific 19) was processed to perform compound‐specific stable carbon and hydrogen isotopic analyses (δ^13^C and δD) of amino acids. For hydroxy acid analysis, an additional 1.236 g of MIL 090001 (parent 48, specific 56) was obtained, while a separate 5.717 g sample of MIL 090001 (specific 41, parent 4) was used for amine studies. For MIL 090657, a total mass of ~1.2 g (parent 12 specific 19) was powdered for analysis. Of this total mass, 153 mg was processed for both amino acid quantification and δ^13^C analyses. An additional 510 mg was used for hydroxy acid studies, and 519 mg was processed for amine analysis.

The meteorite samples, none of which showed any visual evidence of fusion crust, were individually crushed into fine powders and homogenized by mixing using porcelain mortars and pestles (CoorsTek). The MIL 090001 samples were processed in a Class 100 Labconco laminar flow hood under HEPA‐filtered positive pressure at GSFC; the MIL 090657 sample was processed in a Safe‐Aire BSL‐2 cabinet under HEPA‐filtered positive pressure at JSC. Powders were then processed for compound class analyses as described in the following sections. The serpentine samples and procedural reagent blanks were processed in parallel with the meteorite samples as controls.

### Amino Acid Analyses

In order to ensure efficient amino acid extraction, the larger MIL 090001 sample was divided into 18 ampoules containing aliquots of ~1 g each and processed individually until immediately prior to analysis; to each of the ampoules, 4 mL of water was added. For the smaller MIL 090001 and the MIL 090657 samples, 2 mL of water was added. The ampoules were flame‐sealed and placed in an oven at 100 °C for 24 h. After hot water extraction, the test tubes were allowed to cool to room temperature, and then centrifuged for 5 min in a Labconco Centrivap to separate the supernatant from the meteorite grains. The test tubes were opened, and the supernatant was transferred to a clean test tube. The residual meteorite powders were washed twice with 1 mL water and centrifuged for 5 min after the addition of water to each tube. For the MIL 090657 sample and the smaller MIL 090001 sample, half of the extract was set aside for determination of free amino acids. The remaining halves of these two samples, along with each of the 18 larger MIL 090001 samples, were dried under vacuum, and then acid‐vapor hydrolyzed (6 M doubly distilled HCl) for 3 h at 150 °C, to convert acid‐labile amino acid precursors into amino acids. After acid hydrolysis, amino acids in both the hydrolyzed and unhydrolyzed samples were purified by cation‐exchange chromatography using BioRad PolyPrep AG‐50W‐X8 prepacked columns. After removing the caps and snapping off the seals on the Luer tips, the columns were filled to the top with water. Once the volume of water was just above the resin bed, 3 mL of 2M NaOH was added to desorb any contaminating amino acids. The columns were then washed by filling to the top with water twice to remove residual NaOH, after which 3 mL of 1.5 M doubly distilled HCl was added to re‐acidify the column. The columns were again washed with two volumes of water to remove excess HCl. To each sample tube, 10 µL of 25 µM D + L ‐norleucine was added, after which the samples were loaded on the columns. Sample test tubes were rinsed twice with 1 mL water to enable near‐quantitative transfer. The columns were washed with a full column of water. Purified amino acids were eluted by adding two 3.5 mL fractions of 2M NH_4_OH, and then concentrated by vacuum centrifugation.

Immediately prior to analysis, aliquots of the amino acid samples were derivatized with *o*‐phthaldialdehyde and *N*‐acetyl‐L‐cysteine (OPA/NAC) as described previously (Glavin et al. 2006). OPA/NAC amino acid derivatives of the MIL 090001 samples were immediately analyzed with a Waters ACQUITY ultraperformance LC and a Waters ACQUITY fluorescence detector connected in series to a Waters LCT Premier time‐of‐flight mass spectrometer (UPLC‐FD/ToF‐MS) at GSFC. The electrospray and mass spectrometer conditions at GSFC have been described previously (Glavin et al. 2006). The amino acid derivatives of the MIL 090657 sample were analyzed using a Waters ACQUITY H‐Class UPLC and a Waters ACQUITY fluorescence detector connected in parallel to a Waters Xevo G2‐XS quadrupole/time‐of‐flight hybrid mass spectrometer UPLC‐FD/QToF‐MS at JSC. The electrospray and mass spectrometer conditions were positive polarity mode, desolvation gas (N_2_) temperature 500 °C, 1000 L h^−1^; capillary voltage +2.5 kV; cone voltage +40 V, over a mass to charge range of 100–1000 *m*/*z*. Instrument mass calibrations were performed weekly using the Waters Intellistart software, with sodium formate as the reference compound. Mass accuracy measurements were maintained during sample analyses by monitoring the reference compound leucine‐enkephalin (M + H^+^ = 556.2771 Da; 200 pg µL^−1^ concentration with a flow rate of 10 µL min^−1^) for 0.1 s every 30 s. This capillary voltage was also set at +2.5 kV. Both the leucine‐enkephalin and sodium formate solutions were prepared according to Waters directions.

Compound‐specific isotopic measurements were made with a gas chromatograph coupled to a quadrupole mass spectrometer and isotope ratio mass spectrometer (GC‐MS/IRMS) at GSFC. Amino acids were derivatized using isopropanol/trifluoroacetic anhydride. Triplicate injections of derivatized compounds were made in splitless mode in aliquots of 1 μL into a Thermo Trace GC, which was equipped with a 5 m base‐deactivated fused silica guard column (Restek, 0.25 mm ID) and four 25 m length × 0.25 mm ID × 0.25 µm film thickness Chirasil L‐Val capillary columns (Agilent) connected in series using Press‐Tight connectors. The GC was coupled to a Thermo DSQII electron impact quadrupole mass spectrometer and to a Thermo MAT 253 isotope ratio mass spectrometer via a Thermo GC‐C III oxidation interface. Conditions for derivatization, GC‐MS/IRMS analysis, and measurement of δ^13^C values of amino acids have been described previously (Elsila et al. 2012).

### Hydroxy Acid Analyses

MIL 090001 and MIL 090657 were extracted at 100 °C for 24 h inside flame‐sealed glass ampoules. MIL 090001 was extracted in 4 mL of water, while MIL 090657 was separated into ~250 mg portions and extracted in 1 mL of water for each portion. Extracts were separated from residues by centrifugation and transferred to a test tube containing 100 µL of 2 M NaOH. Sample residues were rinsed with water (2 × 0.5 mL) and the rinse supernatants were combined with the aqueous extract. Extracts were dried under vacuum after which 200 µL of DCM, 10 µL of *iso‐*butanol, and 20 µL of boron trifluoride diethyl etherate (≥46.5% BF_3_ basis) were added, and the mixture was heated at 80 °C for 1 h. After cooling to room temperature, 80 µL of acetyl chloride was added to the mixture, which was again heated to 80 °C for 1 h. The reaction mixture was passed through a plug of aminopropyl silica gel and rinsed with ~1 mL of DCM, then blown dry with nitrogen gas, and finally dissolved in ethyl acetate prior to injection (20 or 50 µL).

The derivatized hydroxy acids were analyzed by GC‐MS/IRMS at GSFC using the same instrumentation described for amino acid analyses, with the exception that the four Chirasil L‐Val columns were replaced by four 25 m length × 0.25 mm I.D. × 0.25 µm film thickness CP‐Chirasil‐Dex CB capillary columns (Agilent). The oven program was set as follows: initial temperature was 40°C, ramped at 10 °C min^−1^ to 113 °C, then ramped at 2 °C min^−1^ to 134 °C and held for 8 min, then ramped at 2 °C min^−1^ to 165 °C, and lastly ramped at 10 °C min^−1^ to 210 °C with a final hold of 12 min. The carrier gas used was UHP helium (5.0 grade) at 2.0 mL min^−1^ flow rate. Triplicate injections of derivatized hydroxy acids were made in splitless mode in aliquots of 1 µL. As with amino acids, approximately 10% of the sample eluting from the GC column was directed into a Thermo DSQII electron impact quadrupole mass spectrometer (ion source set at 250 °C and 70 eV, full scan mode from *m/z* 46–400). The mass spectrum was used to identify and quantify the aliphatic hydroxy acids by comparison to reference standards and applying a calibration curve. The remaining 90% of each eluting compound was directed through a Thermo GC‐C III interface for oxidation of the compounds to carbon dioxide after which the δ^13^C stable isotopic measurement was then made on a Thermo MAT 253 IRMS. The δ^13^C values of the eluting compounds were obtained after injection of three pulses of precalibrated CO_2_ (δ^13^C = –24.23‰ Vienna Pee Dee Belemnite [VPDB]) into the IRMS and computation using Thermo Isodat 2.5 software (Figures S1–S2 in supporting information). Conditions for GC‐MS/IRMS analysis and measurement of δ^13^C values have been described in detail previously (e.g., Elsila et al. 2012; Aponte et al. 2014, 2016).

### Amine Analyses

Analyses of amines were performed according to previously published methods (Aponte et al. 2014). Briefly, the samples were separated into ~1 g portions for MIL 090001 and ~250 mg portions for MIL 090657. Each meteorite portion was extracted separately at 100 °C for 24 h inside a flame‐sealed glass ampoule containing 1 mL of 0.1 M HCl. Extract supernatants were separated from residues by centrifugation and transferred to a test tube containing 1 mL of 6 M HCl. Sample residues were rinsed with water (3 × 0.5 mL) and the rinse supernatants were combined with the aqueous acid portion. Extracts were dried under vacuum and subjected to acid vapor hydrolysis using 1 mL of 6 M HCl at 150 °C for 3 h. After hydrolysis, iron hydroxides were precipitated by the addition of 100 µL of 2 M NaOH. The aqueous layer was then separated by centrifugation and the solid residue was rinsed twice with water. The aqueous portions were combined, re‐acidified using 1 mL of 6 M HCl, and dried under vacuum. The residues were re‐dissolved in 2 mL of 1 M NaOH and extracted using DCM (3 × 1 mL). The DCM fractions were combined, passed through a plug of baked anhydrous Na_2_SO_4_, and rinsed once with 0.5 mL of DCM. The organic fractions were treated with 50 mg of functionalized triazabicyclodecene silica gel and 50 µL of 0.1 M (*S*)‐(–)‐*N*‐(trifluoroacetyl)pyrrolidine‐2‐carbonyl chloride (*S*‐TPC, 97% *ee*, Sigma Aldrich). The slurry was stirred for 1 h at room temperature, followed by the addition of 100 mg of aminopropyl silica gel and subsequent stirring for 30 min at room temperature. The mixture was filtered, rinsed with ~3 mL of DCM, dried under nitrogen gas flow, and finally dissolved in ethyl acetate (20 or 50 µL).

The derivatized amines were analyzed by GC‐MS/IRMS using the same instrumentation as was used for the amino acid and hydroxy acid analyses described above (Elsila et al. 2012; Aponte et al. 2014, 2016, 2019). Triplicate injections of derivatized compounds were made in splitless mode using aliquots of 1 μL. The mass spectra were used to identify and quantify compounds through comparison to reference standards run under the same conditions and the application of a 5‐point calibration curve. Conditions for GC‐MS/IRMS analysis and measurement of δ^13^C values have been described previously (Aponte et al. 2014, 2016) (Figures S3–S5 in supporting information).

## Results & Discussion

### Compound Abundances and Isotopic Compositions

**Fig. 1:**
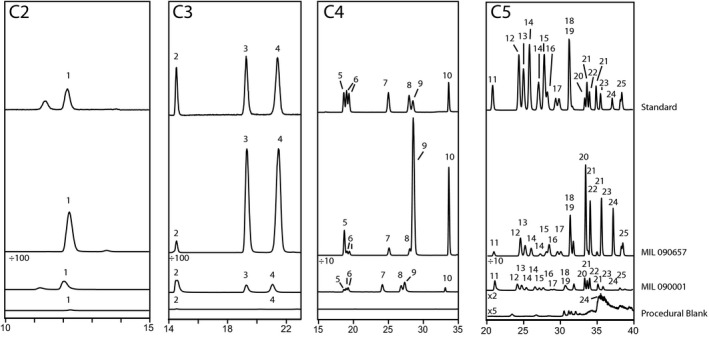
Derivatized samples were separated using a Waters BEH C18 column (2.1 × 50 mm, 1.7 μm particle size) followed in series by a Waters BEH phenyl column (2.1 × 150 mm, 1.7 μm particle size). Chromatographic conditions were: column temperature, 30 °C; flow rate, 150 μL·min^−1^; solvent A (50 mM ammonium formate, 8% methanol, pH 8.0); solvent B (methanol); gradient, time in minutes (%B): 0 (0), 35 (55), 45 (100). Analyses were also performed with the following chromatographic conditions to better resolve the five‐carbon amino acids: column temperature, 30 °C; flow rate, 150 μL·min^−1^; solvent A (50 mm ammonium formate, 8% methanol, pH 8.0); solvent B (methanol); gradient, time in minutes (%B): 0 (15), 25 (20), 25.06 (35), 44.5 (40), 45 (100). The MIL 090001 elution times differ slightly from those of the other samples presented here because the sample was analyzed on a different liquid chromatography system. Peak assignments are: 1) glycine; 2) β‐alanine; 3) D‐alanine; 4) L‐alanine; 5) γ‐amino‐n‐butyric acid; 6) D+L‐β‐aminoisobutyric acid; 7) D‐β‐amino‐n‐butyric acid; 8) L‐β‐amino‐n‐butyric acid; 9) α‐aminoisobutyric acid; 10) D+L‐α‐amino‐*n*‐butyric acid; 11) 3‐amino‐2,2‐dimethylpropanoic acid; 12) 4‐aminopentanoic acid; 13) 4‐amino‐3‐methylpentanoic acid; 14) 3‐amino‐2‐methylbutanoic acid; 15) 3‐amino‐2‐methylbutanoic acid; 16) 5‐aminopentanoic acid; 17) 4‐amino‐2‐methylbutanoic acid; 18) 4‐aminopentanoic acid; 19) 3‐amino‐pentanoic acid; 20) D‐isovaline; 21) 3‐aminopentanoic acid; 22) L‐isovaline; 23) L‐valine; 24) D‐valine; 25) D+L‐norvaline.

We determined the abundances of a suite of ~35 amino acid isomers and enantiomers (25 total species), ranging from two to five carbons, in MIL 090001 and MIL 090657 (Table 1; Figure 1). Table 1 also includes amino acid abundances from other CR chondrites previously analyzed with very similar workup procedures, analytical techniques, and instrumental methods. These meteorites and their petrologic subtypes in the Harju/Rubin scale are Grosvenor Mountains (GRO) 95577 (CR2.0), Graves Nunataks (GRA) 95229 (CR2.7), Elephant Moraine (EET) 92042 (CR2.8), and Queen Alexandria Range (QUE) 99177 (CR2.8). We also measured the abundances of 23 aliphatic hydroxy acid enantiomers and isomers (16 total species), ranging from two to six carbons in length (Table 2; Figures S1 and S2) and 25 aliphatic amines containing up to six carbon atoms present in MIL 090001 and MIL 090657 (Table 3; Figures S3–S5). Table 2 and Table 3 also include measurements of hydroxy acids and amines in the same four additional CR chondrites as shown in Table 1 (Pizzarello et al. 2010; Aponte et al. 2016).

**Table 1:** Summary of the average blank‐corrected amino acid abundances (nmol/g) in the 6 M HCl acid‐hydrolyzed, hot water extracts of CR carbonaceous chondrites.^a^

	GRO 95577 (CR2.0) Glavin et al. (2010)	MIL 090001 (CR2.4) This study	MIL 090657 (CR2.7) This study	GRA 95229 (CR2.7) Martins et al. (2007)	EET 92042 (CR2.8) Glavin et al. (2010)	QUE 99177 (CR2.8) Glavin et al. (2010)
D‐aspartic acid	<0.06	0.19 ± 0.01	8.18 ± 2.66	5.02 ± 0.05	7.72 ± 1.18	4.10 ± 1.61
L‐aspartic acid	<0.02	0.18 ± 0.05	7.90 ± 2.09	5.23 ± 0.07	7.63 ± 1.09	4.03 ± 1.28
D‐glutamic acid	<0.06	0.31 ± 0.11	50.0 ± 11.2	20.4 ± 0.6	68.6 ± 13.4	23.9 ± 4.1
L‐glutamic acid	<0.01	0.25 ± 0.04	46.3 ± 4.3	24.9 ± 2.2	69.2 ± 11.9	23.4 ± 3.7
D‐serine	<0.04	0.05 ± 0.01	3.24 ± 0.37	17.2 ± 0.8	24.6 ± 13.5	6.52 ± 4.05
L‐serine	<0.03	0.06 ± 0.01	2.62 ± 0.32		21.6 ± 11.8	5.79 ± 3.61
D‐threonine	n.r.^e^	<0.01	1.11 ± 0.18	n.r.	n.r.	n.r.
L‐threonine	n.r.	<0.01	0.91 ± 0.17	n.r.	n.r.	n.r.
Glycine	3.7 ± 1.0	3.46 ± 0.88	275 ± 23	770 ± 5	726 ± 205	188 ± 45
D‐alanine	0.52 ± 0.18	0.93 ± 0.23	260 ± 18	569 ± 5	450 ± 104	40.3 ± 5.11
L‐alanine	0.39 ± 0.06	1.06 ± 0.30	267 ± 20	569 ± 32	464 ± 84	39.8 ± 6.7
β‐alanine	3.3 ± 1.1	3.55 ± 0.68	26.0 ± 3.2	32.7 ± 3.1	46.7 ± 13.3	21.9 ± 3.7
D,L‐α‐aminobutyric acid^b^	0.44 ± 0.12	0.63 ± 0.08	107 ± 11	n.r.	202 ± 55	32.0 ± 6.2
D‐β‐aminobutyric acid	0.68 ± 0.10	0.76 ± 0.07	7.14 ± 0.68	58.1 ± 0.8	27.7 ± 5.6	5.67 ± 1.23
L‐β‐aminobutyric acid	0.58 ± 0.09	0.75 ± 0.07	5.92 ± 0.54		30.6 ± 8.3	6.58 ± 1.97
γ‐aminobutyric acid	1.4 ± 0.1	1.09 ± 0.40	22.1 ± 2.9	27.6 ± 1.4	25.4 ± 9.5	12.8 ± 1.1
α‐aminoisobutyric acid	1.7 ± 0.2	1.26 ± 0.12	238 ± 28	268 ± 11	551 ± 151	138 ± 32
D,L‐β‐aminoisobutyric acid^c^	n.r.	2.81 ± 0.37	6.67 ± 1.68	16.0 ± 0.6	n.r.	n.r.
D,L‐norvaline^c^	0.04 ± 0.01	0.09 ± 0.01	8.02 ± 0.71	n.r.	55.5 ± 4.5	16.5 ± 0.8
D‐isovaline	0.12 ± 0.1	0.36 ± 0.05	9.63 ± 0.41	<238	123 ± 5	47.2 ± 1.8
L‐isovaline	0.16 ± 0.02	0.44 ± 0.08	>5.62^g^		121 ± 9	47.5 ± 0.8
D‐valine	0.05 ± 0.01	0.06 ± 0.01	13.6 ± 3.3	49.0 ± 1.8	61.6 ± 3.6	29.4 ± 1.8
L‐valine	0.10 ± 0.01	0.14 ± 0.02	17.9 ± 1.4	51.7 ± 1.3	65.3 ± 4.3	33.0 ± 1.2
D,L‐3‐aminopentanoic acid	0.35 ± 0.03	0.37 ± 0.06	1.50 ± 0.15	n.r.	17.7 ± 1.5	6.86 ± 0.21
D,L‐ and *allo*‐3‐amino‐2‐methylbutanoic acid^c^	0.25 ± 0.02	0.14 ± 0.01	1.85 ± 0.10	n.r.	18.5 ± 0.9	8.21 ± 0.25
3‐amino‐3‐methylbutanoic acid^d^	<0.03	<0.01	<3.15	n.r.	<2.44	<2.13
3‐amino‐2,2‐dimethylpropanoic acid	0.11 ± 0.01	0.19 ± 0.02	0.82 ± 0.05	n.r.	4.60 ± 0.18	2.69 ± 0.17
D,L‐3‐amino‐2‐ethylpropanoic acid^c^	0.75 ± 0.26	0.19 ± 0.04	0.69 ± 0.06	n.r.	11.9 ± 1.1	6.61 ± 0.50
D,L‐4‐aminopentanoic acid^c^	0.64 ± 0.03	0.24 ± 0.02	3.30 ± 0.32	n.r.	25.3 ± 1.8	11.0 ± 0.8
D,L‐4‐amino‐2‐methylbutanoic acid	0.53 ± 0.04	0.07 ± 0.02	3.64 ± 0.33	n.r.	20.5 ± 2.7	12.7 ± 0.5
D,L‐4‐amino‐3‐methylbutanoic acid	0.95 ± 0.20	0.31 ± 0.02	4.28 ± 0.28	n.r.	18.2 ± 0.6	12.6 ± 0.7
δ‐aminovaleric acid	0.33 ± 0.05	0.15 ± 0.02	3.45 ± 0.41	n.r.	8.31 ± 0.79	4.87 ± 0.15
ε‐aminocaproic acid	30. ± 5.	4.11 ± 0.90	>100^g^	n.r.	392 ± 103	19.3 ± 2.4
Total amino acid abundance^f^	17 ± 2	20.1 ± 1.4	1 410 ± 50	2 720 ± 70	3 300 ± 290	790 ± 60

^a^ Values are reported in nanomoles per gram (nmol/g) based on the bulk sample mass. Meteorite extracts were analyzed by OPA/NAC derivatization (15 min) and either LC‐FD⁄ToF‐MS detection or LC‐FD detection for GRA 95229. Monoisotopic chromatograms and fluorescence chromatograms were used for quantification of ToF‐MS data and final peak integrations were background corrected using a procedural blank. Peak areas in the sample chromatograms were compared with pure amino acid standards that were analyzed on the same day. Final values were normalized using desalting and derivatization recoveries based on the D+L‐norleucine internal standard (recoveries were typically 70–80% for the meteorite extracts). Uncertainties (δx) were calculated from the standard error based on the number of separate measurements (n), δ_x_ = σ_x_ n^‐1⁄2^. For all UV fluorescence data, coeluting peaks and⁄or compounds with interfering peaks were not included in the average. Upper limits are presented for amino acids that were not present at levels above the procedural blank background levels.

^b^ Enantiomers could not be separated under the chromatographic conditions.

^c^ Enantiomers could be separated, but not identified, due to a lack of optically pure standards.

^d^ 3‐amino‐3‐methylbutanoic acid coelutes with one of the enantiomers of D,L‐4‐aminopentanoic acid; therefore, upper limits for 3‐a‐3‐mba were estimated by taking the difference in peak areas of the two D,L‐4‐aminopentanoic acid enantiomers.

^e^ n.r. denotes compounds that were not reported in the study.

^f^ The amino acid totals include all compounds in the table except ε‐aminocaproic acid.

^g^ L‐isovaline in this sample co‐eluted with ε‐aminocaproic acid, which was present in sufficiently high abundance to cause ion suppression, precluding accurate quantitation of either compound; therefore, lower limits are reported. Neither compound is included in the total amino acids.

**Table 2:** Abundances of aliphatic hydroxy acids in CR chondrites (nmol/g).^a^

Hydroxy acid	GRO 95577 (CR2.0) Pizzarello et al. (2012)	MIL 090001 (CR2.4) This study	MIL 090657 (CR2.7) This study	GRA 95229 (CR2.7) Pizzarello et al. (2010)	EET 92042 (CR2.8) Pizzarello et al. (2012)	QUE 99177 (CR2.8) Pizzarello et al. (2012)
(*S*)‐α‐lactic acid	484.9^b^	46.5 ± 4.9	16.8 ± 1.1	17.8	131.8^b^	174.0^b^
(*R*)‐α‐lactic acid		46.3 ± 5.4	16.8 ± 1.2	16.8		
glycolic acid	1084.1	40.0 ± 5.1	52.6 ± 4.8	66.7	473.1	156.8
2‐hydroxyisobutanoic acid	34.5	4.9 ± 0.6	5.6 ± 0.7	26.8	109.8	16.8
(*S*)‐2‐hydroxybutanoic acid	49.0^b^	6.2 ± 0.5	2.5 ± 0.2	3.6	19.3^b^	16.8^b^
(*R*)‐2‐hydroxybutanoic acid		6.3 ± 0.7	2.6 ± 0.3	3.3		
(*S*)‐2‐hydroxy‐2‐methylbutanoic acid	21.4^b^	0.8 ± 0.1	3.6 ± 0.2	8.8	2.1^b^	n.f.^c^
(*R*)‐2‐hydroxy‐2‐methylbutanoic acid		0.8 ± 0.1	3.7 ± 0.3	8.6		
(*S*)‐2‐hydroxyisopentanoic acid	9.9^b^	2.8 ± 0.4	1.9 ± 0.1	1.5	3.9^b^	n.f.
(*R*)‐2‐hydroxyisopentanoic acid		2.9 ± 0.5	2.0 ± 0.1	1.5		
(*S*)‐3‐hydroxybutanoic acid	21.4^b^	7.5 ± 1.7	28.9 ± 3.4	4.35	13.2	10.0
(*R*)‐3‐hydroxybutanoic acid^d^		5.9 ± 0.9	19.7 ± 1.0	4.35		
3‐hydroxy‐2,2‐dimethylpropanoic acid	n.r.^e^	2.7 ± 0.5	< 0.02	n.r.^e^	n.r.^e^	n.r.^e^
(*S*)‐3‐hydroxyisobutanoic acid	4.45	4.2 ± 0.7	15.4 ± 1.8	1.5	3.55	1.1
(*S*,*R*)‐2‐hydroxypentanoic acid	31.9	24.3 ± 2.8	1.6 ± 0.1	4.0	1.7	1.5
β‐lactic acid	61.9	3.1 ± 0.4	10.3 ± 0.9	29.5	26.2	18.2
2‐hydroxy‐2‐ethylbutanoic acid	n.r.^e^	< 0.02	< 0.02	n.r.^e^	n.r.^e^	n.r.^e^
(2*S*)‐hydroxy‐(3*S*)‐methylpentanoic acid	n.f.	< 0.02	< 0.02	<0.25	n.f.	n.f.
(2*R*)‐hydroxy‐(3*R*)‐methylpentanoic acid		< 0.02	< 0.02	<0.25		
(*S*,*R*)‐2‐hydroxyisohexanoic acid	11.5	0.6 ± 0.04	< 0.02	<0.5	<1	n.f.
(2*S*,*R*)‐hydroxy‐(3*R*,*S*)‐methylpentanoic acid (diastereomers)	n.f.	< 0.02	< 0.02	<0.5	n.f.	n.f.
(*S*,*R*)‐2‐hydroxyhexanoic acid	23.4	1.3 ± 0.2	< 0.02	n.f.	n.f.	n.f.
Total hydroxy acids abundance	1 840	207 ± 10	184 ± 7	217	785	395

^a^ Values reported in this study are the average of three measurements; errors shown are standard deviations. Errors were not reported for individual compounds in previous studies.

^b^ Data not reported for individual enantiomers.

^c^ n.f. denotes compound not present in sample above detection limits.

^d^ (*R*)‐3‐hydroxyisobutanoic acid co‐elutes with (*R*)‐3‐hydroxybutanoic acid.

^e^ n.r. denotes compound not reported.

**Table 3:** Blank‐corrected concentration for aliphatic amines in the acid‐hydrolyzed hot aqueous extract of MIL 090657 and MIL 090001.^a^

Aliphatic amine	GRO 95577 (CR2.0) Pizzarello et al. (2012)	MIL 090001 (CR2.4) This study	MIL 090657 (CR2.7) This study	GRA 95229 (CR2.7) Aponte et al. (2016)	EET 92042 (CR2.8) Pizzarello et al. (2012)	QUE 99177 (CR2.8) Pizzarello et al. (2012)
*tert*‐butylamine	n.r.^b^	1.1 ± 0.1	2.9 ± 0.2	22.1 ± 0.9	n.r.	n.r.
isopropylamine	n.f.^c^	24.1 ± 4.0	70.9 ± 2.7	570.3 ± 28.9	9.2	17.6
methylamine	43.8	24.4 ± 2.9	394.5 ± 18.8	493.2 ± 12.5	27.7	93.1
dimethylamine	n.f.	< 0.01	57.2 ± 1.2	188.9 ± 14.5	8.2	10.4
ethylamine	n.f.	11.6 ± 1.3	73.6 ± 4.6	186 ± 6	17.9	29.8
*tert*‐pentylamine	n.r.	0.5 ± 0.1	3.4 ± 0.2	11.0 ± 0.5	n.r.	n.r.
methylethylamine	n.f.	2.2 ± 0.3	16.4 ± 1.0	46.6 ± 1.7	<1	2.7
(*R*)‐*sec*‐butylamine	n.f.	0.9 ± 0.1	21.8 ± 1.4	109.0 ± 5.3	0.6	7 ± 1
(*S*)‐*sec*‐butylamine	n.f.	0.9 ± 0.1	20.6 ± 1.2	102.9 ± 4.9	7.9	7 ± 1
diethylamine	n.f.	0.1 ± 0.02	2.8 ± 0.1	12.5 ± 0.5	11.3	18.3
*n*‐propylamine	n.f.	< 0.01	34.0 ± 2.0	127.0 ± 14.7	6.5	11.5
(*R*)‐3‐methyl‐2‐butylamine	n.f.	< 0.01	5.8 ± 0.2	35.3 ± 1.5	3.0	16.5
(*S*)‐3‐methyl‐2‐butylamine	n.f.	n.d.^d^	5.5 ± 0.2	40.8 ± 1.3		
*N*‐methylpropylamine	n.f.	< 0.01	8.4 ± 0.6	15.1 ± 0.3	2.3	4.4
isobutylamine	n.r.	< 0.01	14.1 ± 0.8	13.9 ± 0.5	n.r.	n.r.
(*R*)‐*sec*‐pentylamine	n.f.	n.d.	5.7 ± 0.4	43.1 ± 1.9	5.6	12.5
(*S*)‐*sec*‐pentylamine	n.f.	< 0.01	6.4 ± 0.4	40.9 ± 1.4	1.8	12.5
*N*‐ethylpropylamine	n.r.	n.d.	2.3 ± 0.1	n.d.	n.r.	n.r.
3‐pentylamine	n.f.	n.d.	n.d.	26.9 ± 2.4	6.0	16.1
*n*‐butylamine	n.f.	0.8 ± 0.1	8.1 ± 0.7	10.1 ± 0.3	7.7	71.2
(*R*,*S*)‐2‐methylbutylamine	n.r.	0.3 ± 0.03	4.1 ± 0.1	8.2 ± 0.3	n.r.	n.r.
isopentylamine	n.r.	0.2 ± 0.1	2.4 ± 0.1	3.4 ± 0.04	n.r.	n.r.
*n*‐pentylamine	n.r.	0.2 ± 0.03	2.8 ± 0.2	6.8 ± 0.3	n.r.	n.r.
pyrrolidine	n.r.	0.5 ± 0.1	6.9 ± 0.9	9.4 ± 0.5	n.r.	n.r.
*n*‐hexylamine	n.r.	0.1 ± 0.01	1.1 ± 0.1	1.1 ± 0.04	n.r.	n.r.
Total amine abundance	44	68	772	2 124	120	339

^a^ Compounds identified by comparison with elution time and mass spectra of standards. Values are the average of three measurements from single‐ion gas chromatograms as detailed in the Materials and Methods section; errors shown are standard deviations.

^b^ n.r.: Compound not reported.

^c^ n.f.: Compound not observed above detection limits.

^d^ n.d.: Value could not be determined due to co‐elution (caprolactam, phthalate, or *S*‐TPC reagent) or complex mixture of compounds.

We measured the stable carbon isotope compositions (given as δ^13^C values) for several amino acids in both MIL 090001 and MIL 090657, as well as stable hydrogen isotopic ratios (δD values) for multiple amino acids in MIL 090001 (Table 4). Additionally, we determined the δ^13^C values for several hydroxy acids and amines in each meteorite (Table 5). We also determined the D/L or *R/S* ratios of the chiral amino acids, hydroxy acid, and amines that could be identified and separated (Table 6).

**Table 4:** Stable isotope ratios of amino acids in a range of CR chondrites spanning petrologic types 2.4–2.8.^a^

	MIL 090001 CR2.4 This study		MIL 090657 CR2.7 This study		GRA 95229 CR2.7 Elsila et al. (2012)		EET 92042 CR2.8 Elsila et al. (2012)		QUE 99177 CR2.8 Elsila et al. (2012)	
	δ^13^C	δD	δ^13^C	δD	δ^13^C	δD	δ^13^C	δD	δ^13^C	δD
α‐Amino acids										
D‐glutamic acid	‐	‐	16 ± 2	‐	‐	‐	‐	‐	‐	‐
L‐glutamic acid	‐	‐	18 ± 5	‐	‐	‐	‐	‐	‐	‐
Glycine	10 ± 3	975 ± 128	17 ± 2	‐	35 ± 9	866 ± 60	26 ± 3	1070 ± 82	42 ± 12	1241 ± 216
D‐alanine	11 ± 2	1526 ± 108	16 ± 1^b^	‐	40 ± 3^b^	1382 ± 266	29 ± 2	1693 ± 90	38 ± 10^b^	2204 ± 53^b^
L‐alanine	11 ± 3	2321 ± 101	26 ± 2	‐	38 ± 2	1528 ± 113	34 ± 4	1564 ± 80	39 ± 4	2311 ± 493
α‐aminoisobutyric acid	–3 ± 6	5797 ± 433	–2 ± 4	‐	24 ± 1	4303 ± 1189	25 ± 1	7065 ± 390	20 ± 9	2791 ± 770
D‐α‐amino‐*n*‐butyric acid	5 ± 2	‐	5 ± 3^b^	‐	20 ± 3	1920 ± 204	20 ± 2	2769 ± 557	‐	‐
L‐α‐amino‐*n*‐butyric acid	‐	‐	4 ± 5	‐	10 ± 14	2099 ± 263	22 ± 3	3409 ± 232	–8 ± 4	1945 ± 59
Isovaline	‐	‐	–9 ± 3	‐	16 ± 8	3813 ± 253	21 ± 2	6017 ± 395	7	4956 ± 57
D‐valine	‐	‐	7 ± 2	‐	18 ± 3	2165 ± 307	16 ± 10		‐	‐
L‐valine	‐	‐	–6 ± 2^b^	‐	13 ± 7	2086 ± 345	21 ± 5	3307 ± 283	3 ± 12	1822 ± 95
non‐α‐Amino acids										
β‐alanine	–6 ± 2	2835 ± 353	8 ± 7^b^	‐	8 ± 20	2286 ± 367	18 ± 9	3462 ± 70	–1 ± 6	2253 ± 112
γ‐amino‐*n*‐butyric acid	–21 ± 4	‐	‐	‐	–18 ± 11	‐	5 ± 3	2018 ± 105	–10 ± 4	736 ± 39

^a^ Values are the average of three measurements from single‐ion gas chromatograms as detailed in the in Materials and Methods section; errors shown are standard deviations of three injections.

^b^ Includes small contribution from unidentified coeluting peak.

**Table 5:** δ^13^C values (‰VPDB) for unhydrolyzed aliphatic hydroxy acids and acid‐hydrolyzed aliphatic amines in MIL 090657 and MIL 090001

Aliphatic hydroxy acid	MIL 090001 (CR2.4)^a^	MIL 090657 (CR2.7)^a^
Glycolic acid	9 ± 1	46 ± 2
(*S*)‐α‐lactic acid	–12 ± 2	–2 ± 3
(*R*)‐α‐lactic acid	–14 ± 2	1 ± 2
(*S*)‐2‐hydroxybutanoic acid	1 ± 2	n.d.
(*R*)‐2‐hydroxybutanoic acid	–1 ± 2	n.d.
(*S*)‐2‐hydroxyisopentanoic acid	–8 ± 1	n.d.
(*R*)‐2‐hydroxyisopentanoic acid	–10 ± 2	n.d.
(*S*,*R*)‐2‐hydroxypentanoic acid	–15 ± 2	n.d.
β‐lactic acid	0 ± 5	8 ± 3
Aliphatic amine		
*tert*‐butylamine	n.d.	n.d.
isopropylamine	–19 ± 3	–13 ± 3
methylamine	–8 ± 6	15 ± 13
dimethylamine	–46 ± 9	n.d.
ethylamine	–16 ± 9	5 ± 4
methylethylamine	n.d.	–10 ± 2
(*R*)‐*sec*‐butylamine	n.d.	–6 ± 5
(*S*)‐*sec*‐butylamine	n.d.	–3 ± 7
*n*‐propylamine	–31 ± 3	–10 ± 8

n.d.: Value could not be determined due to co‐elution or complex mixture of compounds.

^a^ Values are the average of three measurements from single‐ion gas chromatograms as detailed in the Materials and Methods section; errors shown are standard deviations of three injections.

**Table 6:** D/L or *R*/*S* ratios of chiral amino acids,^a^ hydroxy acids,^b^ and amines^b^ found in MIL 090001 and MIL 090657

Amino acids (D/L)	MIL 090001 D/L or *R*/*S*	MIL 09001 %L_ee_ or %*S*_ee_ ^c^	MIL 090657 D/L or *R*/*S*	MIL 090657 %L_ee_ or %*S*_ee_
aspartic acid	1.06 ± 0.30	−2.7 ± 14.5	1.04 ± 0.43	−1.7 ± 21.3
glutamic acid	1.24 ± 0.48	−11 ± 22	1.08 ± 0.26	−3.8 ± 12.6
serine	0.83 ± 0.22	9.1 ± 11.9	1.24 ± 0.21	−11 ± 9
threonine	n.d.^d^	n.d.	1.22 ± 0.30	−10 ± 14
alanine	0.88 ± 0.33	6.5 ± 17.6	0.97 ± 0.10	1.3 ± 5.0
valine	0.43 ± 0.09	40 ± 7	0.76 ± 0.19	−9.3 ± 7.2
β‐aminobutyric acid	1.01 ± 0.13	−0.7 ± 6.6	1.21 ± 0.16	−9.3 ± 7.2
isovaline	0.83 ± 0.31	10 ± 10	n.d.	n.d.
Hydroxy acids (*R*/*S*)				
lactic acid	1.00 ± 0.16	−0.2 ± 7.9	1.00 ± 0.10	0.0 ± 4.8
2‐hydroxybutanoic acid	1.02 ± 0.14	−0.8 ± 6.8	1.04 ± 0.15	−2.0 ± 6.9
2‐hydroxy‐2‐methylbutanoic acid	1.00 ± 0.18	0.0 ± 8.7	1.03 ± 0.10	−1.4 ± 4.8
2‐hydroxyisopentanoic acid	1.04 ± 0.23	−1.8 ± 11.4	1.05 ± 0.08	−2.6 ± 3.7
Amines (*R*/*S*)				
*sec*‐butylamine	1.00 ± 0.16	0.0 ± 7.8	1.06 ± 0.09	−2.8 ± 4.5
3‐methyl‐2‐butylamine	–	–	0.95 ± 0.05	−2.7 ± 2.6
*sec*‐pentylamine	–	–	0.89 ± 0.08	5.8 ± 4.4

^a^ Errors for amino acid D/L ratios were calculated from propagation of the standard errors for each compound, σ_x_ = x([σ_a_/a]^2^ + [σ_b_/b]^2^)^1/2^

^b^ Values have been corrected against injections of racemic standards to account for instrument response. Enantiomeric ratios and standard deviations of amines are based on three separate measurements from single‐ion gas chromatograms extracted at *m/z* = 165.5–168.5 for *sec*‐butylamine, and at *m/z* = 235.5–238.5 for 3‐methyl‐2‐butylamine and *sec*‐pentylamine.

^c^ %L_ee_ values are reported for the averaged amino acid total abundances based on the formula, ([L‐D]/[L + D]×100%), where negative values denote a D‐excess; similarly, *S*_ee_ values were calculated from the averaged hydroxy acid and amine abundances using the formula ([*S*‐*R*]/[*S* + *R*]×100%), with negative values indicating an *R*‐excess.

^d^ D/L ratios were not calculated for threonine in MIL 090001 because it was not present above detection limits nor for isovaline in MIL 090657 because of a compound co‐eluting with L‐isovaline that prevented accurate quantitation.

### Assessing the Extent of Terrestrial Contamination

Several lines of evidence suggest that most or all of the amino acids, hydroxy acids, and amines detected in both MIL 090001 and MIL 090657 are indigenous to the meteorites and not the result of terrestrial contamination. Because each of these compound classes has a different potential contamination signature, each class will be discussed separately.

Chiral protein amino acids that are ubiquitous in terrestrial biology, and are thus most likely to be potential contaminants, typically have D/L ratios <0.3 when measured with our analytical procedures (Burton et al. 2011, 2014a; Onstott et al. 2014). In the meteorite samples, however, most of the protein amino acids that are present have D/L enantiomeric ratios that are nearly equal to 1 (Table 6), suggesting that the amino acids detected in the meteorite are not from biological sources. The two meteorites also possess a wide range of amino acids that are rare or nonexistent in terrestrial biology, also indicating a non‐biological origin. A third line of evidence supporting an extraterrestrial origin for many of the amino acids is the stable isotope composition of these compounds. Biosynthesis of all 20 α‐amino acids found in terrestrial proteins begins from metabolic intermediates of glycolysis and the citric acid cycle, meaning the isotopic signatures of the biological amino acids are driven by isotopic fractionation during photosynthesis and subsequent enzymatic reactions (Scott et al. 2006). In the case of carbon, the vast majority of biological amino acids have δ^13^C values less than 0‰, although a few amino acids have been found in certain organisms to have values up to + 30‰. For hydrogen, δD compositions are largely driven by the deuterium compositions of water sources, which typically fall between 0 and –90‰, and are then further depleted during amino acid biosynthesis (Fogel et al. 2016). In biological organic compounds, δD values range from +50 to –370‰. The δD values for the amino acids that were measured in MIL 090001 (glycine, D‐ and L‐alanine, β‐alanine, and α‐aminoisobutyric acid) clearly confirm an extraterrestrial origin, ranging from +975‰ to +5797‰ (Table 4). The δ^13^C values for glycine, and D‐ and L‐alanine, are also consistent with an extraterrestrial origin (+10‰, +11‰, and +11‰, respectively), although these values are lower than previously reported for other weakly altered CR chondrites of similar petrographic types (Elsila et al. 2012). The δ^13^C values for α‐aminoisobutyric acid, β‐alanine, and γ‐amino‐*n*‐butyric acid (–3‰, –6‰, and –21‰, respectively), however, would be ambiguous on their own, illustrating a limitation of δ^13^C measurements alone for definitively establishing an extraterrestrial origin of putative meteoritic amino acids (Burton et al. 2012a; Callahan et al. 2013; Glavin et al. 2020). For α‐aminoisobutyric acid and β‐alanine in MIL 090001, this is not an issue because their δD values are also clearly extraterrestrial, but for γ‐amino‐*n*‐butyric acid, where δD measurements could not be made due to low abundances and chromatographic interferences, it is less clear‐cut. A previous δ^13^C measurement of γ‐amino‐*n*‐butyric acid extracted from terrestrial soil gave a value of –31 ± 3‰ (Burton et al. 2014a), suggesting that the MIL 090001 γ‐amino‐*n*‐butyric acid could be extraterrestrial, a conclusion that is supported by the observation that the bulk of the amino acids observed in this meteorite are clearly extraterrestrial in origin. A broader suite of amino acids was analyzed for δ^13^C in MIL 090657, but we did not have enough available sample mass after amino acid, hydroxy acid, and amine analyses to perform δD measurements of the amino acids. Nevertheless, the majority of the individual amino acids measured for δ^13^C in MIL 090657 have distinctive extraterrestrial compositions (+16‰ to +26‰). A notable exception to this trend is L‐valine (–6 ‰ in MIL 090657). Considering that this amino acid had a lower D/L ratio (0.76) than the other proteinogenic amino acids, it is likely that there was some contribution of terrestrial L‐valine contamination in this sample. We were unable to determine a specific source for the L‐valine contamination, but it does not appear to be from proteins, which would be expected to contribute all 20 of the proteinogenic amino acids. If one were to assume that all of the L‐valine enantiomeric excess (~4.3 nmol g^−1^) were the result of contamination, based on the average frequency of amino acid occurrence in proteins (Kozlowski 2017), one would expect similar contamination levels from amino acids of similar abundance in proteins, including threonine and serine. However, threonine and serine were actually racemic within error in MIL 090657, and the L‐enantiomer abundances of each were significantly less than would be expected from extrapolation of L‐valine contamination (0.91 and 2.62 nmol g^−1^, versus ~4.3 nmol g^−1^, respectively).

Terrestrial contamination by hydroxy acids would plausibly be predominated by *S*‐lactic acid (denoted L by analogy to amino acids). It is ubiquitous in biology as it is produced during glycolysis, and is subsequently converted to other compounds (ethanol, acetic acid, glycerol, etc.) during anaerobic fermentation, or converted back into glucose under aerobic conditions (Smith et al. 1975). In contrast, although *R*‐lactic acid (D‐lactic acid by analogy to amino acids) is produced during the detoxification of methylglyoxal in many organisms, and is a normal metabolite for some fungi and bacteria, it is present in significantly lower abundances than *S*‐lactic acid (Lee et al. 2013). The simplest hydroxy acid, glycolic acid, is produced by plants during photorespiration and from the detoxification of glyoxal (Lee et al. 2013; Maurino and Engqvist 2015). Both glycolic acid and lactic acid are also increasingly used for the industrial production of biodegradable polymers such as for drug delivery (e.g., Makadia and Siegel 2011). Glycolic acid, *R*‐lactic acid, and *S*‐lactic acid were found in nearly equal abundance (1:1:1) in MIL 090001, whereas these same hydroxy acids are present in a ratio of 1:0.3:0.3 in MIL 090657. Both of these distributions differ markedly from what would be expected for terrestrial contamination, specifically a predomination of *S*‐lactic acid, due to its ubiquity in metabolism. Biodegradable polymers also seem to be an unlikely source of contamination because they are designed to not persist in the environment (Arcos‐Hernandez et al. 2012). Furthermore, for each of the hydroxy acids where we were able to measure both enantiomers without any interferences, the enantiomeric composition was racemic (*R/S* ≈ 1) within the margin of error (Table 6), as would be expected from abiotic synthesis. This observation, coupled with the apparent lack of terrestrial contamination observed for the amino acids in the two meteorites, supports the conclusion that the observed hydroxy acids are indigenous. In contrast with the stable isotope measurements of amino acids, the δ^13^C values of hydroxy acids are of limited utility for distinguishing between extraterrestrial and terrestrial origins of hydroxy acids. Previously, Pizzarello and co‐workers showed that hydroxy acids from Murchison with δ13C values ranging from −20.4 ‰ to +13.3 ‰ had δD values of +406 ‰ to +2395 ‰, establishing a clear extraterrestrial origin (Pizzarello et al. 2010). Although the δ^13^C values for hydroxy acids in both MIL 090001 and MIL 090657 reported here fall in the same range as the carbon isotope compositions measured in Murchison, in the absence of δD values for the same compounds, δ13C values alone do not appear sufficient for establishing an extraterrestrial origin for hydroxy acids given the current state of knowledge.

Aliphatic amines on Earth may come from several anthropogenic sources such as cattle feedlot operations, car exhaust, waste incineration, and sewage treatment (Mosier et al. 1973; Cadle and Mulawa 1980; VandenBoer et al. 2011). Thus, just as with amino and hydroxy acids, meteorite contamination with aliphatic amines could occur. However, unlike amino acids and hydroxy acids, amines may be lost quickly after impregnation based on their lower vapor pressures and reactivity (Hoshika 1977; Audunsson and Mathiasson 1984; Roberts et al. 1988). Aliphatic amines such as mono‐, di‐, and trimethylamine are some of the most common bacterial degradation products (Zeisel et al. 1983, 1985; Al‐waiz et al. 1987), as are aliphatic diamines such as putrescine and cadaverine, and thus are ubiquitous in biology. However, aliphatic straight‐chained monoamines with more than three carbon atoms are not as abundant in the terrestrial biosphere, with typical atmospheric abundances on the order of ppt_v_ [ng m^−3^] (Audunsson and Mathiasson 1983; Grönberg et al. 1992a, 1992b; Chang et al. 2003; VandenBoer et al. 2011, 2012). While the enantiomeric composition of *sec*‐butylamine and other chiral aliphatic amines present in the terrestrial biosphere remains to be measured, the racemic nature of aliphatic amines such as *sec*‐butylamine (Table 6) in MIL 090657 and MIL 090001 suggests that the amines we found are indigenous from the meteorite samples and not the result of terrestrial contamination. As with hydroxy acids, the δ^13^C values measured for the amines in MIL 090001 and MIL 090657, on their own, are not sufficient to distinguish between an extraterrestrial or terrestrial origin for these compounds.

### Abundances and Structural Distributions

Despite both MIL 090001 and MIL 090657 being nominally classified as CR2 chondrites, MIL 090657 contained approximately 70‐fold higher amino acid abundances than MIL 090001. Compared with other CR chondrites analyzed by the same methods for the same suite of amino acids (Glavin et al. 2010), the total abundances of amino acids in MIL 090657 (CR2.7; ~1400 nmol g^−1^) are comparable to those found in several other weakly altered CR2.7/2.8 chondrites (790–3000 nmol g^−1^; Table 1). In contrast, the total amino acid abundance of ~20 nmol g^−1^ in MIL 090001 (CR2.4) is very similar to that of the CR2.0 chondrite Grosvenor Mountains (GRO) 99577 (~17 nmol g^−1^).

The structural distributions of amino acids within these samples also follow similar trends. The ratio of β‐alanine to glycine in GRO 95577 (CR2.0) and MIL 090001 (CR2.4) is 0.9 and 1.0, respectively, versus 0.09–0.11 in MIL 090657 (CR2.7), EET 92042 (CR2.8), and QUE 99177 (CR2.8). The ratio of β‐alanine to glycine was previously observed to correlate with the degree of parent body aqueous alteration (Glavin et al. 2010), an observation that would suggest that MIL 090001 experienced significantly more aqueous alteration than did MIL 090657. This is consistent with their relative alteration sequences on the Harju and Rubin scale, in the order GRO 95577 (2.0) < MIL 090001 (2.4) < MIL 090657 (2.7) < EET 92042 (2.8) ~ QUE 99177 (2.8), as well as mineralogical classification based on secondary alteration phases (Alexander et al. 2013; Harju et al. 2014; Davidson et al. 2015; Howard et al. 2015; Abreu 2016). MIL 090001 has also been described as an anomalous or thermally altered CR2.4 (Harju et al. 2014), and was originally given a classification of CV2 (Keller 2011; Keller et al. 2012; Alexander and Bowden 2018). It is possible that the anomalous alteration history of MIL 090001 is responsible for differences observed between its organic molecule contents and those of the other CR chondrites.

**Fig. 2:**
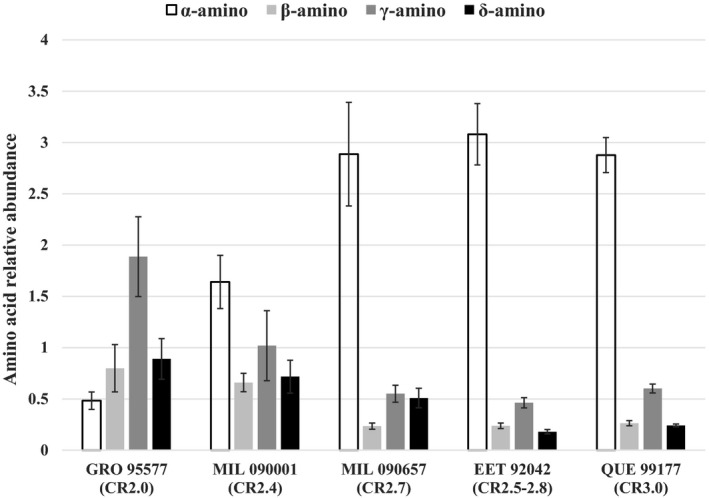
Comparison of C5 amino acid distributions among CR chondrites. Petrographic types indicated under each meteorite name are from the Alexander and Rubin scales (Abreu 2016; Alexander et al. 2013; Harju et al. 2014; Davidson et al. 2015; Howard et al. 2015).

The five‐carbon (C5) amino acid distributions, which have also been shown to vary predictably with parent body alteration (Glavin et al. 2010; Burton et al. 2012a), are consistent with MIL 090001 having experienced more aqueous alteration than MIL 090657 (Fig. 2). MIL 090657, in particular, contains predominantly α‐amino acids like EET 92042 and QUE 99177. MIL 090001 has significantly more β‐, γ‐, and δ‐amino acids, though it still has a higher relative abundance of α‐amino acids overall, giving it an intermediate distribution between GRO 95577 and the other three CR chondrites (Fig. 2).

The total abundances of the hydroxy acids in MIL 090001 and MIL 090657 (Table 2) are comparable to those measured previously for GRA 95229, but two‐ to ten‐fold lower than the hydroxy acid abundances reported for EET 92042, QUE 99177, and GRO 95577 (Pizzarello et al. 2008, 2012). The most abundant hydroxy acids in each of the six CR chondrites are glycolic acid and (*S*)‐ and (*R*)‐α‐lactic acid. Notably, however, in MIL 090001, MIL 090657, and GRA 95229, these compounds comprise 47–64% of the total hydroxy acid abundances, whereas in GRO 95577, EET 92042, and QUE 99177, they represent ~77 to 85% of the hydroxy acids present. The order of magnitude variation in total hydroxy acid abundances among the six meteorites with a broad range of aqueous alteration is much smaller than the ~190‐fold variation observed in amino acid abundances (Table 1).

The total abundances of aliphatic amines (Table 3) measured in MIL 090657 and MIL 090001 in the current study are lower than those reported in the CR2.7 chondrite GRA 95229, similar to those observed in EET 92042 (CR2.8) and QUE 99177 (CR2.8), and higher than the level in the CR2.0 GRO 95577. Amines in MIL 090657 are half as abundant as in GRA 95229, while amines in MIL 090001 are over an order of magnitude lower in concentration. Another particularity between the amine compositions of MIL 090657 and MIL 090001 and previously examined CR2 chondrites is the varying abundances of methylamine with respect to isopropylamine. In MIL 090001, methylamine and isopropylamine are found in similar concentrations. In MIL 090657, methylamine is more abundant than isopropylamine, as was also previously found to be the case in EET 92042 (CR2.8) and QUE 99177 (CR2.8) (Pizzarello et al. 2012). These distributions all differ from GRA 95229 (CR2.7), where the abundance of isopropylamine is greater than that of methylamine (Table 3; Aponte et al. 2016). The variability in amine distribution in these CR chondrites may suggest that the CR parent body had a heterogeneous accretion, and/or that processes such as radionuclide and shock heating, as well as aqueous alteration, shaped the abundances of amines in their parent body (note that these factors would also affect the abundances of amino acids and hydroxy acids as well, though not necessarily to the same degree for each compound class). However, notable similarities in the amine molecular distributions between these CR chondrites are also seen, including a decrease in amine concentration with increasing carbon number, and a preponderance of aliphatic amines having the amino moiety (–NH_2_) in a secondary carbon over isomers having the amino group on a primary carbon or tertiary carbon in C_3_‐C_5_ amines (Aponte et al. 2016). These molecular similarities point toward common synthetic pathways for meteoritic amines in the CR parent body.

### Enantiomeric Excesses

As seen in Table 6, most of the chiral compounds analyzed in the MIL 090001 and MIL 090657 CR2 chondrites are present in racemic mixtures, within experimental uncertainty. The L‐valine excesses observed in both MIL 090001 and MIL 090657 may be an indication of some terrestrial contamination, particularly when combined with the lighter δ^13^C value for L‐valine compared to D‐valine in MIL 090657. The uncertainty in the D/L ratio for isovaline in MIL 090001 precludes a firm declaration of an L‐isovaline excess, although L‐isovaline was present in L‐excess in four of the five sample injections, with an average L‐excess of 10.0% ± 4.0% across all five measurements when the enantiomeric excess was calculated for each sample run prior to averaging (individual enantiomeric excess values were: 8.7%, 17.6%, 4.9%, 20.2%, −1.6%). This value is consistent with previous observations of L‐isovaline excesses in aqueously altered CM and CR chondrites (Glavin et al. 2010). The excess we see in the more altered MIL 090001 is greater than that observed in the CR2.8 EET 92042 (−1%) and CR2.8 QUE 99177 (~0%), and comparable to that found in the CR2.0 GRO 95577 (~11%). Combined with previous analyses showing correlations between greater amounts of aqueous alteration and increased L‐enantiomeric excesses (Glavin et al. 2010), this observation further supports the finding that MIL 090001 experienced greater aqueous alteration than EET 92042. We also observed a D‐excess in β‐amino‐*n*‐butyric acid above analytical uncertainty in MIL 090657. Across three sample injections, β‐amino‐*n*‐butyric acid was always found in D‐excess, with an average excess of 8.7% ± 2.0%, when enantiomeric excess was calculated for each sample run and then averaged (6.6%, 9.0%, and 10.6% were the individual enantiomeric excesses per run). This is an intriguing result as it would mark the first observation of an extraterrestrial enantiomeric excess in a non‐α‐amino acid isomer outside analytical uncertainty, as the only such previous report was 7% ± 10% (Koga and Naraoka 2017). We were able to measure the ^13^C composition of both D‐ and L‐β‐amino‐*n*‐butyric acid in MIL 090657, and found that the δ^13^C values were equal to each other (5 ± 3 ‰ and 4 ± 5 ‰, respectively) and consistent with amino acids of extraterrestrial origin. One caveat to this is that β‐amino‐*n*‐butyric acid has been reported to be a natural metabolite of plants. However, the chiral distribution was not determined, and the abundances were orders of magnitude lower than for protein amino acids, which would be expected to also be present as contaminants (Thevenet et al. 2017). Consequently, this compound is an interesting target for future studies of meteoritic amino acids. No other D‐enantiomeric excesses of amino acids with a single asymmetric carbon have been reported in a meteorite (Glavin et al. 2020). The racemic nature of the lactic acid observed in MIL 090001 and MIL 090657 is in contrast to the slight (3–12%) excesses previously reported for *S*‐lactic acid in the GRA 95229 and LAP 02342 CR2 chondrites (Pizzarello et al. 2010); however, the previous analyses did not report experimental uncertainties, so the exact magnitude of any enantiomeric excess is unknown. Terrestrial contamination was also suggested as a potential source of the *S*‐lactic acid in the previous study (Pizzarello et al. 2010). Our racemic measurements of the other chiral hydroxy acids measured in MIL 090001 and MIL 090657 (2‐hydroxybutanoic acid, 2‐hydroxy‐2‐methylbutanoic acid, and 2‐hydroxyisopentanoic acid) are the first enantiomeric analyses of these compounds in meteorites. Similarly, the chiral amines measured in our work are also racemic (Table 6). This is consistent with previous measurements of chiral amines in multiple CM and CR2 chondrites (Aponte et al. 2014, 2016, 2014, 2016).

### Isotopic Ratios

As described above, the majority of the amino acids in both MIL 090001 and MIL 090657 showed enrichments in both ^13^C and D compared to terrestrial values (Table 4). The amino acids glycine, D‐alanine, and L‐alanine were more ^13^C‐enriched in MIL 090657 than in MIL 090001, which in turn were lower than in other weakly altered CR chondrites (Elsila et al. 2012), while two others (α‐aminoisobutyric acid and D‐α‐amino‐*n*‐butyric acid) had similar δ^13^C values in the two meteorites. For each of the four hydroxy acids whose isotopic compositions were measured in both meteorites, the hydroxy acids in MIL 090657 were more enriched in ^13^C than the same molecules isolated from MIL 090001 (Table 5). Similarly, amines in MIL 090657 were enriched in ^13^C relative to those in MIL 090001 (Table 5). The isotopic values for amines measured in MIL 090657 were within the range of values measured previously in other CR chondrites (Aponte et al. 2016). The lower δ^13^C values in MIL 090001 compared to MIL 090657 may be related to the unusual petrologic characteristics of MIL 090001 relative to other CR chondrites or its more extensive history of aqueous alteration and/or thermal processing.

A decrease in δ^13^C with increasing number of carbons was observed for straight‐chained C_1_‐C_3_ amines in MIL 090657; a similar correlation has been seen for amines in CM2 chondrites such as LON 94101 and LEW 90500 (Aponte et al. 2016). In contrast, no clear correlation between δ^13^C and carbon content was observable in MIL 090001, a characteristic consistent with previous δ^13^C measurements of other CR chondrites such as GRA 95229 and LAP 02342 (Aponte et al. 2016). These two divergent relationships between δ^13^C and carbon chain length may suggest that chain elongation of aliphatic amines occurred through more than a single preferred synthesis or destruction mechanism, or that it was influenced by differences among available precursor materials during parent body processing.

It is possible that stable isotope ratios could potentially be used to help elucidate whether hydroxy acids and amino acids are formed from the same precursors and in the same environments. For example, if alanine and lactic acid were both formed via Strecker‐cyanohydrin chemistry, or by the reduction of α‐keto acids in the presence of ammonia from acetaldehyde and cyanide, they would be expected to have similar isotopic compositions and experience similar levels of fractionation from the addition of cyanide. However, we did not observe any clear correlations between the isotopic signatures of structural analogs of the compound classes measured in this work. Overall, amines and hydroxy acids were less isotopically enriched than amino acids. However, a more concerted effort beyond the measurements reported here must be undertaken, as was done for amino acids (Elsila et al. 2012), to enable robust conclusions to be drawn about the potential formation relationships of these compounds.

### Compound Class Variations with Aqueous Alteration

Previous amino acid analyses of CR chondrites revealed that amino acid abundances decreased with increasing aqueous alteration, while the distribution of amino acids tended to increase in favor of the non‐α‐amino acids, which may be related to their enhanced resistance to decarboxylation (Glavin et al. 2010). Hydroxy acids appear to be more abundant in the samples that experienced more extensive aqueous alteration (Pizzarello et al. 2012). Amine abundances and distributions did not seem to show any clear trends that correlated with CR petrologic type (Pizzarello et al. 2012). It is possible that the syntheses of these compounds in meteorite parent bodies may be linked. For example, amino acids where the amino group is located on the carbon adjacent to the carboxylic acid moiety (α‐amino acids) may be formed from the same precursor pool as α‐hydroxy acids with Strecker‐cyanohydrin chemistry (Miller 1957; Peltzer et al. 1984). Also, amines may have been formed through the degradation of amino acids by decarboxylation (Li and Brill 2003; McCollom 2013; Pietrucci et al. 2018).

We compared the ratios of hydroxy acids, amino acids, and amines among CR chondrites based on aqueous alteration (Table 7). Interestingly, the ratio of hydroxy acids to amino acids varies more than 1,000‐fold among the meteorites studied, with the most aqueously altered meteorites (GRO 95577 and MIL 090001) having ratios greater than 10:1 and the remaining, less aqueously altered meteorites having hydroxy acid to amino acid ratios around 1:10 (Table 7). The hydroxy acid to amine ratios are less variable, though only available for three of the chondrites. Nevertheless, again we see higher ratios in the more altered meteorite (MIL 090001) compared to the less altered ones (MIL 090657 and GRA 95229). Because this is a small data set, additional meteoritic measurements of these compound classes are required to understand whether this type of variation consistently correlates with aqueous alteration or if it is more dependent on individual differences between meteorites.

**Table 7:** Comparison of the abundances of amino acids, hydroxy acids, and amines in CR chondrites determined from a common suite of compounds measured for each meteorite

Compounds	GRO 95577 (CR2.0)	MIL 090001 (CR2.4)	MIL 090657 (CR2.7)	GRA 95229 (CR2.7)	EET 92042 (CR2.8)	QUE 99177 (CR2.8)
Total amino acids (nmol/g)	17.3^a^	20.1	1410	2722^b^	3300^a^	790^a^
Total hydroxy acid (nmol/g)	1840^c^	207	184	217^d^	695^c^	395^c^
Total amines (nmol/g)	n.a.	67.8	771.8	2124.2^e^	n.a.	n.a.
						
Hydroxy acids ÷ amino acids	106.4	10.3	0.1	0.1	0.2	0.5
Amino acids ÷ amines	n.a.	0.3	1.8	1.3	n.a.	n.a.
Hydroxy acids ÷ amines	n.a.	3.1	0.2	0.1	n.a.	n.a.

n.a.: Not available.

^a^ Glavin et al. (2010)

^b^ Pizzarello et al. (2008)

^c^ Pizzarello et al. (2012)

^d^ Pizzarello et al. (2010)

^e^ Aponte et al. (2016).

## Conclusions

We have analyzed the abundances, distributions, and enantiomeric and isotopic compositions of amines, amino acids, and hydroxy acids in two previously unstudied CR chondrites, MIL 090001 and MIL 090657. Combined with existing data in the literature, similar suites of these compounds have now been analyzed in a set of six CR chondrites, spanning the range of aqueous alteration types from 2.0 to 2.8 on the Harju and Rubin petrologic scale. Of the three compound classes, amino acids were the most sensitive to variation in petrologic type, with abundances varying nearly 200‐fold, whereas amines varied 50‐fold and hydroxy acids only showing a 10‐fold variation. Nearly all of the chiral compounds that were clearly extraterrestrial in origin were present as racemic mixtures, with L‐isovaline (~10% excess) and D‐β‐amino‐*n*‐butyric acid (~9% excess) as the notable exceptions. Further isotopic studies are needed to verify the chiral excess of D‐β‐amino‐*n*‐butyric acid. The isotopic compositions of compounds reported here did not reveal any definitive links between the different compound classes but may provide a foundation and framework for further in‐depth analyses in the future.

## Editorial Handling

Dr. Scott Sandford

## Supporting information


**Figure S1.** Structures of studied aliphatic amines.
**Figure S2.**Positive electron impact GC‐MS chromatogram (23–40 min region, *m/z*= 101+115+129) and typical GC‐IRMS chromatogram obtained at *m/z*= 44 (12CO2 peak) during carbon compound‐specific isotope analysis of hot water extracted derivatized hydroxy acids from MIL 090657, MIL 090001, procedural blank, and commercially available standards (all traces are on the same intensity scale, except for the standard trace).
**Figure S3.**Structures of studied aliphatic amines.
**Figure S4.**Positive electron impact GC‐MS chromatogram (22–48 min region, *m/z*= 166) and typical GC‐IRMS chromatogram obtained at *m/z*= 44 (12CO2 peak) during carbon compound‐specific isotope analysis of hot water extracted *S*‐TPC‐derivatized amines from MIL 090001, procedural blank, and commercially available standards (all traces are on the same intensity scale, except for the standard trace).
**Figure S5.**Positive electron impact GC‐MS chromatogram (22–48 min region, *m/z*= 166) and typical GC‐IRMS chromatogram obtained at *m/z*= 44 (12CO2 peak) during carbon compound‐specific isotope analysis of hot water extracted *S*‐TPC‐derivatized amines from MIL 090657, procedural blank, and commercially available standards (all traces are on the same intensity scale, except for the standard trace).
