# Supplementary material for: Analysis of amino acids, hydroxy acids, and amines in CR chondrites
Source: Meteorit Planet Sci. 2020 Dec 16;55(11):2422–39. doi: 10.1111/maps.13586 (PMC7839561; doi:10.1111/maps.13586)
Supplement: Supplementary file 1 — Figure S1. Structures of studied aliphatic amines. Figure S2.Positive electron impact GC‐MS chromatogram (23–40 min region, m/z= 101+115+129) and typical GC‐IRMS chromatogram obtained at m/z= 44 (12CO2 peak) during carbon compound‐specific isotope analysis of hot water extracted derivatized hydroxy acids from MIL 090657, MIL 090001, procedural blank, and commercially available standards (all traces are on the same intensity scale, except for the standard trace). Figure S3.Structures of studied aliphatic amines. Figure S4.Positive electron impact GC‐MS chromatogram (22–48 min region, m/z= 166) and typical GC‐IRMS chromatogram obtained at m/z= 44 (12CO2 peak) during carbon compound‐specific isotope analysis of hot water extracted S‐TPC‐derivatized amines from MIL 090001, procedural blank, and commercially available standards (all traces are on the same intensity scale, except for the standard trace). Figure S5.Positive electron impact GC‐MS chromatogram (22–48 min region, m/z= 166) and typical GC‐IRMS chromatogram obtained at m/z= 44 (12CO2 peak) during carbon compound‐specific isotope analysis of hot water extracted S‐TPC‐derivatized amines from MIL 090657, procedural blank, and commercially available standards (all traces are on the same intensity scale, except for the standard trace). [file MAPS-55-2422-s001.docx]

**Supporting Information**

Analysis of amino acids, hydroxy acids and amines in CR chondrites.

José C. Aponte^1,2^, Jamie E. Elsila^2^, Jason E. Hein^3^, Jason P. Dworkin^2^, Daniel P. Glavin^2^, Hannah L. McLain^1,2^, Eric T. Parker^2^, Timothy Cao^3^, Eve L. Berger^4^, Aaron S. Burton^5*^

^1^Department of Chemistry, Catholic University of America, Washington, D. C. 20064; ^2^Solar System Exploration Division, NASA Goddard Space Flight Center, Greenbelt, MD 20771; University of British Columbia, British Columbia, Canada; Texas State University / Jacobs JETS contract, Astromaterials Research and Exploration Science Division, NASA Johnson Space Center, Houston, TX 77058; ^5^Astromaterials Research and Exploration Science Division, NASA Johnson Space Center, Houston, TX 77058

* To whom correspondence should be addressed: [aaron.burton@nasa.gov](mailto:aaron.burton@nasa.gov)

**Figure S1.** Structures of studied aliphatic amines.

**Figure S2.** Positive electron impact GC-MS chromatogram (23-40 min region, *m/z* = 101+115+129) and typical GC-IRMS chromatogram obtained at *m/z* = 44 (^12^CO_2_ peak) during carbon compound-specific isotope analysis of hot-water extracted derivatized hydroxy acids from MIL 090657, MIL 090001, procedural blank, and commercially available standards (all traces are on the same intensity scale, except for the standard trace). Unidentified peaks represent compounds that could not be adequately identified or analyzed because of co-elutions or insufficient sensitivity. Compound identifications: **1**, (*S*)-lactic acid; **2**, 2-hydroxyisobutyric acid; **3**, (*R*)-2-lactic acid; **4**, glycolic acid; **5**, (*S*)-2-hydroxybutyric acid; **6**, (*R*)-2-hydroxybutyric acid; **7**, (*S*)-2-hydroxy-2-methylbutyric acid; **8**, (*R*)-2-hydroxy-2-methylbutyric acid; **9**, (*S*)-2-hydroxyisopentanoic acid; **10**, (*R*)-2-hydroxyisopentanoic acid; **11**, (*S*)-3-hydroxybutyric acid; **12**,**13**, (*R*)-3-hydroxybutyric acid/(*R*)-3-hydroxyisobutyric acid; **14**, 3-hydroxy-2,2-dimethylpropanoic acid; **15**, (*S*)-3-hydroxyisobutyric acid; **16**, (*S*,*R*)-2-hydroxypentanoic acid; **17**, β-lactic acid; **18**, 2-hydroxy-2-ethylbutyric acid; **19**, (2*S*)-hydroxy-(3*S*)-methylpentanoic acid (diastereomers); **20**, (2*R*)-hydroxy-(3*R*)-methylpentanoic acid (diastereomers); **21**, (*S*,*R*)-2-hydroxyisohexanoic acid; **22**, (2*S*,*R*)-hydroxy-(3*R*,*S*)-methylpentanoic acid (diastereomers); **23**, (*S*,*R*)-2-hydroxyhexanoic acid; **U**: unknown compound.

**Figure S3.** Structures of studied aliphatic amines.

**Figure S4.** Positive electron impact GC-MS chromatogram (22-48 min region, *m/z* = 166) and typical GC-IRMS chromatogram obtained at *m/z* = 44 (^12^CO_2_ peak) during carbon compound-specific isotope analysis of hot-water extracted *S*-TPC-derivatized amines from MIL 090001, procedural blank, and commercially available standards (all traces are on the same intensity scale, except for the standard trace). Unidentified peaks represent compounds that could not be adequately identified or analyzed because of co-elutions or insufficient sensitivity. Compound identifications: **1**, *tert*-butylamine; **2**, isopropylamine; **3**, methylamine; **4**, dimethylamine; **5**, ethylamine; **6**, *tert*-pentylamine; **7**, methylethylamine; **8**, (*R*)-*sec*-butylamine; **9**, diethylamine; **10**, (*S*)-*sec*-butylamine; **11**, *n*-propylamine; **12**, (*R*)-3-methyl-2-butylamine; **13**, *N*-methylpropylamine; **14**, isobutylamine; **15**, (*R*)-*sec*-pentylamine; **16**, (*S*)-3-methyl-2-butylamine; **17**, *N*-ethylpropylamine; **18**, 3-pentylamine; **19**, (*S*)-*sec*-pentylamine; **20**, *n*-butylamine; **21**, (*R*,*S*)-2-methylbutylamine; **22**, isopentylamine; **23**, *n*-pentylamine; **24**, pyrrolidine; **25**, *n*-hexylamine. **P**: phthalate, **R**: reagent (*S*-TPC acid), **U**: unknown compound.

**Figure S5.** Positive electron impact GC-MS chromatogram (22-48 min region, *m/z* = 166) and typical GC-IRMS chromatogram obtained at *m/z* = 44 (^12^CO_2_ peak) during carbon compound-specific isotope analysis of hot-water extracted *S*-TPC-derivatized amines from MIL 090657, procedural blank, and commercially available standards (all traces are on the same intensity scale, except for the standard trace). Unidentified peaks represent compounds that could not be adequately identified or analyzed because of co-elutions or insufficient sensitivity. Compound identifications: **1**, *tert*-butylamine; **2**, isopropylamine; **3**, methylamine; **4**, dimethylamine; **5**, ethylamine; **6**, *tert*-pentylamine; **7**, methylethylamine; **8**, (*R*)-*sec*-butylamine; **9**, diethylamine; **10**, (*S*)-*sec*-butylamine; **11**, *n*-propylamine; **12**, (*R*)-3-methyl-2-butylamine; **13**, *N*-methylpropylamine; **14**, isobutylamine; **15**, (*R*)-*sec*-pentylamine; **16**, (*S*)-3-methyl-2-butylamine; **17**, *N*-ethylpropylamine; **18**, 3-pentylamine; **19**, (*S*)-*sec*-pentylamine; **20**, *n*-butylamine; **21**, (*R*,*S*)-2-methylbutylamine; **22**, isopentylamine; **23**, *n*-pentylamine; **24**, pyrrolidine; **25**, *n*-hexylamine. **P**: phthalate, **R**: reagent (*S*-TPC acid), **U**: unknown compound.
